# App-Based Intervention Combining Evidence-Based Behavior Change Techniques With a Model-Based Reasoning System to Promote Physical Activity Among Young Adults (Active2Gether): Descriptive Study of the Development and Content

**DOI:** 10.2196/resprot.7169

**Published:** 2018-12-21

**Authors:** Anouk Middelweerd, Saskia J te Velde, Julia S Mollee, Michel CA Klein, Johannes Brug

**Affiliations:** 1 VU Medical Center Amsterdam Department of Epidemiology & Biostatistics Amsterdam Netherlands; 2 te Velde Research & Consultancy Bunnik Netherlands; 3 VU University Amsterdam Department of Computer Science Amsterdam Netherlands; 4 University of Amsterdam Faculty of Social and Behavioural Sciences Amsterdam Netherlands

**Keywords:** physical activity, mHealth, moderate-vigorous physical activity, mobile phones

## Abstract

**Background:**

The Active2Gether intervention is an app-based intervention designed to help and encourage young adults to become and remain physically active by means of personalized, real-time activity tracking and context-specific feedback.

**Objective:**

The objective of our study was to describe the development and content of the Active2Gether intervention for physical activity promotion.

**Methods:**

A systematic and stepwise approach was used to develop the Active2Gether intervention. This included formulating objectives and a theoretical framework, selecting behavior change techniques, specifying the tailoring, pilot testing, and describing an evaluation protocol.

**Results:**

The development of the Active2Gether intervention comprised seven steps: analyzing the (health) problem, developing a program framework, writing (tailored) messages, developing tailoring assessments, developing the Active2Gether intervention, pilot testing, and testing and evaluating the intervention. The primary objective of the intervention was to increase the total time spent in moderate-vigorous physical activity for those who do not meet the Dutch guideline, maintain physical activity levels of those who meet the guideline, or further increase physical activity levels if they so indicated. The theoretical framework is informed by the social cognitive theory, and insights from other theories and evidence were added for specific topics. Development of the intervention content and communication channel resulted in the development of an app that provides highly tailored coaching messages that are framed in an autonomy-supportive style. These coaching messages include behavior change techniques aiming to address relevant behavioral determinants (eg, self-efficacy and outcome expectations) and are partly context specific. A model-based reasoning engine has been developed to tailor the intervention with respect to the type of support provided by the app, send relevant and context-specific messages to the user, and tailor the graphs displayed in the app. For the input of the tailoring, different instruments and sensors are used, such as an activity monitor (Fitbit One), Web-based and mobile questionnaires, and the location services on the user’s mobile phone.

**Conclusions:**

The systematic and stepwise approach resulted in an intervention that is based on theory and input from end users. The use of a model-based reasoning system to provide context-specific coaching messages goes beyond many existing eHealth and mHealth interventions.

## Introduction

Insufficient physical activity (PA) is a risk factor for avoidable burden of disease [1,2]. About 25% of the adult population worldwide [2] and around 50% in many western countries such as the US and the Netherlands [3] do not meet the recommended guidelines for PA. Moreover, engagement in moderate-vigorous PA (MVPA) decreases with age, in particular when transitioning from adolescence into (young) adulthood [4,5].

In general, health promotion interventions informed by established health behavior theory have been found to be associated with higher effect sizes than interventions not based on theory [6-8]. Research examining the determinants of PA mainly focuses on social cognitive and social ecological factors [9-15]. Social cognitive theories and models, such as the health belief model [16], the theory of planned behavior [17], and the social cognitive model [13], have been developed to explain health behaviors and guide health behavior research and behavior change [18,19]. Although these models mainly focus on intrapersonal and interpersonal factors, social ecological models more explicitly recognize that behavior may also be strongly influenced by contextual factors, such as the sociocultural and physical environments people live in [19-21]; for example, Sallis et al [20] proposed a framework recognizing that individuals are physically active within different domains (eg, recreation, transport, household, and occupation), where different factors on multiple levels influence their overall PA behavior. Thus, interventions that aim to increase levels of PA should not only target intra- and interpersonal factors but also take their physical and social environments into account.

Besides interventions being informed by theory, interventions are more likely to be effective when established behavior change techniques (BCTs) are incorporated [6,8,22]. More specifically, interventions that included a self-monitoring feature in combination with features such as prompting intention formation, specific goal setting, providing feedback on performance, or reviewing behavioral goals were significantly more effective at promoting PA and healthy eating than interventions that did not include these BCTs [8].

Systematic reviews further showed that Information and communications technology (ICT)-supported, individually tailored interventions are superior to generic interventions in promoting PA and user engagement and appreciation [6,23-25]. Moreover, Krebs et al [23] demonstrated that dynamic tailoring (ie, iteratively assessing and providing feedback) was associated with larger effect sizes than static tailoring (ie, all feedback is based on one baseline assessment) [23]. Additionally, Rabbi et al [26] reported promising results when using machine-learning techniques to automatically create contextualized and personalized feedback to increase levels of PA. Modern technology, such as smartphones, smartphone apps, and activity trackers, offer new possibilities in health promotion, especially in young adults, of whom the majority owns a smartphone [27,28]. Furthermore, the rapid growth of the popularity and variety of health and fitness apps and activity trackers suggest that young adults will appreciate and adopt an app-based PA intervention.

Several content analyses have been conducted to identify if and how constructs of behavior change theories and BCTs are incorporated in PA promotion apps. Generally, the apps analyzed were lacking applications of behavior change theories and the use of evidence-based BCTs [29-33]. Moreover, apps mostly provide generic advice or tips about PA; gamification, punishment, and context-aware feedback are rare among PA apps [34]. Only a few apps incorporate some form of adaption to the user [34]. Lastly, existing apps fail to meet the guidelines for PA [35,36]. Despite the fact that health and fitness apps are popular among smartphone users [37,38], recent research indicates that most presently available apps lack the necessary empirical basis to make a meaningful difference in PA promotion [7]. Thus, those apps are less likely to be effective, and room for improvement exists when using an app to promote physical activity. A recently published systematic review examined studies that used apps in interventions to influence health behavior, including PA [39]. The majority of those studies that targeted adults reported significant short-term intervention effects on levels of PA [39]. Furthermore, the majority of the interventions that reported significant changes in behaviors and health-related outcomes included BCTs, such as goal setting, self-monitoring, and feedback on the performance [39].

In summary, innovative ICT-supported mobile technology-based approaches that are evidence based and include dynamic tailoring using intelligent data interpretation techniques may help to effectively support achievement and maintenance of behavior change in the PA domain. However, both the empirical basis and dynamic tailoring are lacking in current apps. Thus, PA apps that incorporate constructs of behavior change theories and BCTs and provide dynamically tailored feedback are needed. Therefore, we developed the Active2Gether intervention that combines mobile (app-based) technology with dynamically tailored feedback and aims to go beyond existing (mobile) PA interventions. The Active2Gether intervention is an app-based intervention designed to help and encourage young adults to become and remain physically active by focusing on the domains of active transport, stair climbing, and sports participation. To do so, participants of the Active2Gether intervention will be categorized into one of the 3 awareness categories (education, coaching, and feedback). Participants in the education category will receive educational messages on the benefits of PA, whereas participants in the feedback category will receive motivational messages to maintain their active lifestyle. Participants who are in the coaching category will be coached on sports participation, taking the stairs, or active transport. Every week, the participants will be asked to choose their coaching domain and to set a weekly goal. Participants will receive a message with a suggestion for a coaching domain and a weekly goal based on their previous behavior, but the final decision will be up to the user. The participants will receive a Fitbit One activity tracker that can be synced to the Active2Gether app and will allow the participants to monitor their PA behavior through the Active2Gether app. Additionally, participants will receive (daily) coaching messages addressing relevant behavioral determinants. The content of the messages will be tailored to the user’s behavioral determinants, occupational status, and weather. Lastly, the intervention offers the opportunity to monitor and compare the behaviors with those of other Active2Gether participants because the app will display the activity data of the participant, including a graph displaying the activity data of 6 other participants, preferably friends. The graph with the activity data of others will rank the participants based on their step activity and the user preferences for social comparison (ie, upward or downward comparison). Taking this preference into account does influence the effectiveness of social comparison as a behavior change technique [40]. The aim of this paper was to describe the systematic development and content of this Active2Gether PA-promotion intervention. The methods section provides a brief overview of the stepwise approach that was used to develop the intervention and a brief description of the target population and the methodology used to develop the intervention. The results section will provide more detailed information on the results of the systematic development and content of the intervention.

## Methods

### Target Population

The Active2Gether intervention focuses on healthy and highly educated young adults aged 18-30 years who have a suitable smartphone running on Android version 4.0 or higher.

### Intervention Development

We used a 7-step systematic approach to develop and evaluate the intervention (Table 1). To ensure that the app was informed by relevant health behavior and health behavior change theory and evidence, the development was guided by the program-planning model developed by Kreuter et al [41]. Some steps were adapted because it felt more logical to the research team, and the order of some steps were changed; for example, creating tailoring algorithms, automating the tailoring process, and developing the communication channel are described in the same step. The 7 steps are further described in the Results sections.

**Table 1:** Description of the stepwise process for the development of Active2Gether.

Steps	Step description
Step 1: Analyzing the (health) problem	Describing a theoretical framework on how to promote MVPA^a^ Selecting behavior change techniques based on theory and evidence to address determinants of behavior, based on existing studies and reviews [42,43]; Assessing existing apps (what is available?) [30]; Exploring preferences of end users [44,45]
Step 2: Developing a Program Framework	Identifying relevant physical activity behaviors to increase MVPA. Defining the main and subobjectives of the intervention Describing framework components
Step 3: Writing (tailored) messages (the order of this step was changed: Step 5)^b^	Writing tailored messages
Step 4: Developing tailoring assessments (the order of this step was changed: Step 3)^b^	Selecting and developing measurements to assess levels of physical activity, behavioral determinants, locations, and connected friends
Step 5: Developing the Active2Gether intervention (steps were merged)^c^	Designing tailoring algorithms for the reasoning system Channel of communication: building a Web-based app and system to combine and interpret data and send messages
Step 6: Pilot testing	Pilot-testing the intervention to detect errors and impracticalities in order to improve the intervention prior to its implementation
Step 7: Testing and evaluating the intervention	The intervention will be used by a larger group of participants and then analyzed and evaluated with respect to effect, process, and impact

^a^MVPA: moderate-vigorous physical activity.

^b^According to the program-planning model by Kreuter et al [41], the tailored messages should be written in step 5, whereas the tailoring assessments should be developed in step 3.

^c^Creating tailoring algorithms, automating the tailoring process, and developing the communication channel are described in the same step, whereas according to the program-planning model, these are steps 6 and 7, respectively.

### Step 1: Analyzing the (Health) Problem

#### Identifying Determinants of Change and Reviewing Applicable Theories and Models

Because theory-based interventions are associated with higher effect sizes than interventions not based on theory [6,46], defining the theoretical framework for the intervention is necessary. To do so, prominent health behavior theories and scientific literature were reviewed.

**Figure 1:**
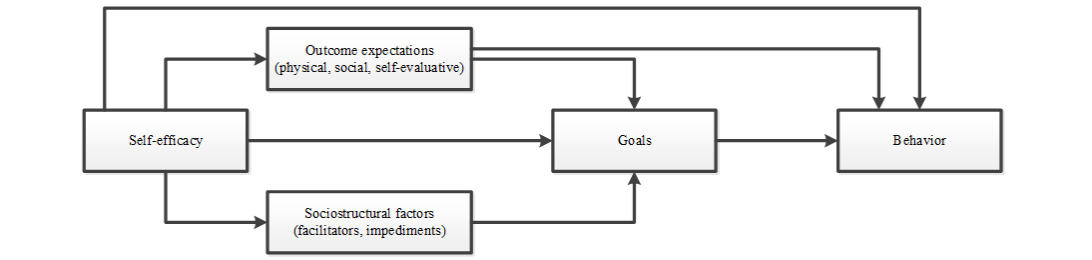
The structural pathways of Bandura’s social cognitive theory.

**Figure 2:**
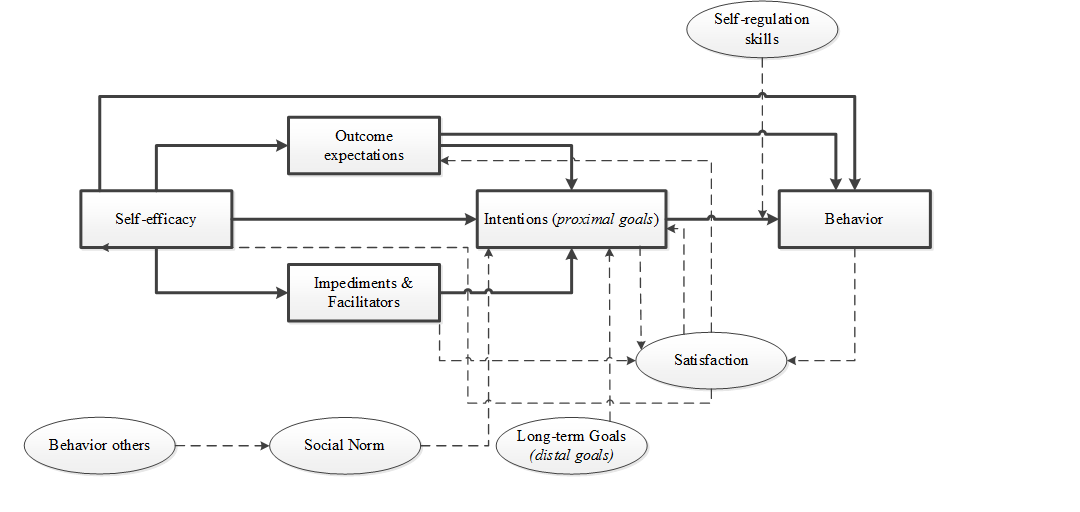
The specific theoretical framework that is used for the Active2Gether intervention.

Social cognitive theory (SCT) was adopted as a basis for the theoretical framework as it is one of the most prominent behavior change theories used to inform interventions targeting health behavior change [10,47,48], and a recent meta-analysis reported that SCT concepts may explain 31% of variance in PA [10]. SCT addresses both individual and social factors and recognizes the reciprocal relation between individuals and their context or environment. For these reasons, SCT thus guided and informed the intervention’s theoretical framework; insights from other theories and evidences were added for specific topics. Figure 1 shows the structural pathways of Bandura’s SCT [49], and Figure 2 shows the specific theoretical framework [49] that is used for the Active2Gether intervention. In Figure 2, the bold lines and boxes represent the elements that are based on the Social Cognitive Theory, and the dotted lines and oval boxes represent behavioral determinants added to the theoretical framework.

#### Selection of Behavior Change Techniques

We first identified evidence-based and relevant BCTs and linked these with the behavioral determinants of the theoretical framework by means of a review of the relevant literature, based on an existing taxonomy of BCTs [42,43]; see Multimedia Appendix 1 [9,22,42,43,50-53]. To explore which BCTs were used in already existing PA promotion apps, a systematic content analysis of such apps available in iTunes and Google Play was conducted [30]. Additionally, focus group discussions with the target population were conducted. The methods and results of these focus groups have been published in more detail elsewhere [45]. Finally, a Web-based cross-sectional survey was conducted among 179 young adults to assess their ratings with respect to the importance of specific BCTs applied in apps and their preferences for personalized tailoring [44].

### Step 2: Developing a Program Framework

#### Defining the Intervention’s Primary and Secondary Objectives

The foundation of the intervention is the definition of the program’s outcomes and objectives [54,55]. Therefore, specifying who and what will change as a result of the intervention is necessary [54,55]. Intervention objectives were based on the Dutch guidelines for physical activity for adults, which included the following: 30 minutes of moderate PA for at least 5 days a week or 20 minutes of vigorous PA for 3 days a week [56].

#### Describing Framework Components

Based on steps 1 and 2 and the research team’s expertise, a general framework was developed. The aim was to develop a highly tailored intervention that contains a self-monitoring tool, goal setting, social comparison, and motivational and context-specific messages.

Tailoring and personalization of the intervention content is realized in the following 6 ways: determining the personally appropriate type of support (ie, education, coaching, or feedback), selecting the personally relevant and preferred domain of PA for user coaching (ie, sports participation, stair use, or active transport), suggesting a weekly goal, selecting the personally appropriate behavioral determinants for coaching, sending only relevant coaching messages and filtering out nonrelevant messages, and tailoring and personalization of the app content.

### Step 3: Writing (Tailored) Messages

Next, we translated the BCTs into actual tailored feedback messages and advice. For each BCT per behavioral determinant (Multimedia Appendix 1), a set of messages was created that was tailored to the three coaching domains (ie, sports participation, active transport, and stair climbing). Consequently, a message library was created that contained feedback and advice messages tailored to all possible levels of the relevant behavioral determinants, as recognized in the underlying theoretical framework.

Creating the message library was an iterative process of brainstorming, writing a set of messages (AM), and providing feedback and suggestions (JM and StV). To test whether the tone of voice and content appealed to the target population, a subset of messages was pilot-tested among 7 people of the target population.

### Step 4: Developing Tailoring Assessments

To tailor the messages to the individual users, assessment methods were selected.

#### Assessment of Activity

First, after considering functionalities, validity, and costs of a range of available activity trackers, the user’s activity was monitored using Fitbit One, which includes monitoring of steps and stairs climbed. Fitbit One was chosen because of its functionalities and small size [57]. The activity monitor communicates with the Fitbit app and website that display the collected data for example by showing a color-coded chart indicating the proximity to the step goal, which is set to a default of 10,000 steps per day.

Fitbit allows developers and researchers to access Fitbit data and thus integrate the Fitbit data into health behavior interventions such as Active2Gether. To access Fitbit data, Fitbit offers an application programming interface (API). Fitbit One was validated using smaller time intervals (ie, minutes, hours, and days) relevant for real-time feedback and instant behavioral insights to its users. Healthy young adults (N=34) wore the ActiGraph GT3x+ and a Fitbit One for one week. Detailed information on the methodology can be found elsewhere [58].

#### Assessment of Behavioral Determinants

Literature was reviewed for relevant, existing, and validated questionnaires to assess behavioral determinants. Behavioral determinants are assessed by means of a questionnaire with both its long and short versions, which were selected based on validations of such questionnaires. The long version is part of an “intake” questionnaire before the actual intervention and as a point of departure for the tailored intervention, whereas the short version is used repeatedly throughout the intervention period to dynamically tailor the intervention content to the user.

#### Assessment of Location Data

A questionnaire was designed for the purpose of assessing information on significant places. In addition, the Active2Gether app was built in a way that enabled the collection of the user’s location data.

#### Assessment of Connected Friends

To increase the users’ engagement, we assessed whether users’ friends were also participating in the Active2Gether intervention. Because Facebook is very popular among Dutch young adults—93% of Dutch adults aged 18-24 use Facebook [59]—Facebook was used to find connected friends that were also participating in the study.

### Step 5: Developing the Active2Gether Intervention

#### Creating Tailored Algorithms

To realize such tailored coaching, we developed a system that combines detailed behavior monitoring with intelligent data interpretation and model-based predictions. Thus, by combining data from the different sources, the system enables personalization of the coaching strategies to try to achieve the most positive effect on behavior change. Detailed information on the system and the development of the system can be found elsewhere and is not described in the Results section [60].

#### Designing and Developing the Communication Channel

We decided that the communication channel of the Active2Gether intervention should be a smartphone app. The app shows the website in a format that is viewable on smaller screens. Thus, the intervention content was accessible through the app or the website. The research team developed the design template of the smartphone app. Information on the development of the app can be found elsewhere [60].

### Step 6: Pilot Testing

To detect possible bugs in the system and to assess user friendliness and appreciation, the app was pilot-tested in two steps. First, the Active2Gether team (AM, JSM, Adnan Manzoor Rajper, SJtV, and MCAK) used the initial version of the Active2Gether app. Bugs, nuisances, etc, were monitored, listed, and fixed accordingly when and where possible. Second, 7 people from the target population (5 women, 21-28 years old, all highly educated, or studying at the bachelor’s or master’s level) were recruited to use the adjusted version of the app, monitor bugs and nuisances, provide feedback in person, and answer a questionnaire regarding use, user friendliness, and appreciation. The app was further adjusted based on that information.

### Step 7: Testing and Evaluating the Intervention

The intervention, the Active2Gether app, will be evaluated for its efficacy to change weekly levels of MVPA in young adults and for the usability of the app.

## Results

### Step 1: Analyzing the (Health) Problem

#### Identifying Determinants of Change and Reviewing Applicable Theories and Models

As a result of Step 1, a theoretical framework was built based on the relevant scientific literature (please see further details below). The theoretical framework was subsequently used to develop the content of the intervention and predict the PA behavior of the users so that the intervention content could be tailored to each individual user.

Self-efficacy, a key construct within SCT (and in other health behavior theories) [18,49], was adopted as a key construct in Active2Gether. Self-efficacy is defined as someone’s beliefs in his or her own capabilities to perform certain actions needed to achieve a desired outcome. Self-efficacy affects PA both directly and indirectly, as seen in Figures 1 and 2. Self-efficacy may influence outcome expectations—one’s beliefs about the positive and negative consequences of one’s behavior, such as participating in physical activities [18,49]. In other words, people who are more efficacious about being physically active will also be more likely to expect the favorable outcomes of participating in physical activities. [49] Moreover, self-efficacy may also influence how people perceive potential obstacles and impediments [49] and may also influence intentions to engage in PA behaviors [61]. Goal setting was adopted as a second important basis for change, where goals can be either proximal (ie, shorter-term intentions to act) or distal (ie, longer-term goals to achieve something) [49,62]. Distal goals are goals set for the longer term and they set the course for personal change [62]. According to Bandura [49], distal or long-term goals can initiate behavior change but are not sufficient to change behavior directly, as seen in Figure 1. Goal setting is dependent on the levels of self-efficacy and perceived barriers and opportunities. In line with this notion, a meta-analysis inspired by the action-control framework indicated that 48% of the participants who intended to be physically active failed to do so. Therefore, forming intentions is often not sufficient to realize behavior change; self-regulatory and action-control techniques are needed to support behavioral enactment [63]. A further meta-analysis on effective techniques in healthy eating and PA interventions concluded that interventions that offered self-monitoring and addressed self-regulation were more successful in increasing PA than interventions not including those techniques [8]. SCT posits that when individuals adapt and revise their behavior, they may adjust their beliefs and goals regarding this behavior [49]. In our theoretical framework, we therefore included “satisfaction,” which is defined as an evaluation of the PA behavior.

In line with SCT, we also recognized that the social environment influences behavior through social norms and that performing certain behaviors can evoke social reactions, both positive and negative [49]. In the Active2Gether intervention, we address not only intrapersonal (eg, lack of motivation and tiredness) and social barriers (eg, lack of support) but also contextual impediments (eg, lack of time, weather and travel distance), as seen in Figure 2. Lastly, it was decided that users will be categorized based on their awareness of their personal PA levels before they will be coached; people who are overly optimistic about their PA levels (ie, who believe they engage in adequate amounts of PA while their data show insufficient levels) will be much less likely to be motivated to increase their PA levels [64].

#### Selection of Behavior Change Techniques

Content analysis showed that the apps available to date generally lack sufficient incorporation of evidence-based BCTs [30]. BCTs that were applied most often were providing feedback on performance, prompting self-monitoring of behavior, prompting specific goal setting, and planning social support or social change [30]. Additionally, focus group discussions with the target population indicated that participants preferred self-monitoring, goal setting, and a ranking feature but were not willing to share their accomplishments on social media for social comparison and initiating social support [45]. The focus groups further suggested that the Active2Gether app should be highly personalized, have an easy-to-use design and format, include a coaching feature that provides tailored feedback to self-set goals, enable competition with friends by ranking or earning rewards, and include the option to personally customize the application [45]. Finally, a Web-based cross-sectional survey among 179 young adults to assess their ratings with respect to the importance of specific BCTs applied in apps and their preferences for personalized tailoring confirmed the need for a personal coaching feature and showed that BCTs addressing goal setting, goal reviewing, feedback, and self-monitoring were rated as important to be incorporated in an app, whereas social support and social comparison were considered less important [44]. The combined results of the literature review, focus group discussions, and survey guided the selection of BCTs to be included in Active2Gether (Multimedia Appendix 1).

### Step 2: Developing a Program Framework

#### Defining the Intervention’s Primary and Secondary Objectives

Step 2 resulted in the decision to make the following the primary objective of the Active2Gether intervention: increase total time spent in MVPA for those who do not meet the Dutch guideline, maintain PA levels of those who meet the guideline, or further increase PA levels if they so indicated. The secondary aims were defined as follows: to increase the underlying specific categories of MVPA (ie, minutes of weekly sports participation, weekly numbers of stairs climbed, and weekly minutes of active transport) and to enhance the underlying determinants of the PA behaviors.

#### Describing Framework Components

The framework contained information on the levels of tailoring and an outline of the steps taken to deliver tailored messages. Detailed information on the framework components can be found in Multimedia Appendix 2.

### Step 3: Writing (Tailored) Messages

In line with Self-Determination Theory [65], the messages were written in an autonomy-supportive style. Messages were also written in a way that supports relatedness and individualization (eg, by addressing the users personally by their names). By respecting their autonomy and making them feel related to the Active2Gether intervention, we aimed to increase the user’s willingness to follow up on the coaching messages. Moreover, the messages were written in a positive gain-framed style, that is, a style that describes the potential gains (eg, in health, fitness, and relaxation) when participating in PA rather than focusing on loss (ie, ill health, lack of fitness, and stress) when not engaging in PA [66]. The majority of the messages were tailored to determinants in the theoretical framework, the weather, and occupational status.

A pilot test of a subset of messages among 7 female bachelor’s and master’s students indicated that the messages were friendly, motivational, and empathic; some were perceived as autocratic, whereas some were not. Some minor changes were made to the messages.

### Step 4: Developing Tailoring Assessments

Further decisions were made on how to measure the characteristics for tailoring messages.

#### Assessment of Physical Activity

Our test of the validity of the Fitbit One indicated that Fitbit can be considered a valid device to assess step activity for real-time minute-by-minute self-monitoring, although an overestimation of 677 steps per day by Fitbit was seen compared with the ActiGraph [58]. However, the validation study indicated that Fitbit is less suitable for providing instant real-time feedback and daily feedback on PA intensity levels (ie, minutes of moderate, vigorous, or MVPA) because it substantially and systematically overestimates the time spent per intensity level per hour [58]. For that reason, Fitbit is only used to assess step activity.

Participants need to give permission once for the application to access their activity data. These then can be collected regularly, and a summarized version of the data is stored in the Active2Gether database. These data are utilized in the following several ways: for presenting the activity level (ie, number of steps and number of stairs climbed) to the user, for determining the type of coaching, and for tailoring coaching messages.

#### Assessment of Behavioral Determinants

We decided to assess behavioral determinants by means of a questionnaire with both its long and short versions, which were selected based on the validations of such questionnaires. The long version is based on existing questionnaires that have previously been validated (ie, Neighborhood Quality of Life Survey and Self-efficacy scales) or questions used in previous studies and were translated and adapted where necessary [20,21,67]. In the short questionnaire, we decided to use single item questions to assess each of the behavioral determinants that are part of the framework and the system. In the short version of the questionnaire, all determinants are specified for each coaching domain (ie, sports participation, stairs use, and active transport). These items were not pretested as such but were based on the long questionnaire. Multimedia Appendix 3 [12,68,69] provides an overview of the questions asked in the long and short versions of the questionnaire, including the answer options.

#### Assessment of Location Data

We also included questions about the participants’ significant places (eg, home address, parental home, sports location, university, work location) in the intake questionnaire. These questions focus on travel options from their home to significant locations, thus information about the active and nonactive transportation options. Additionally, information about the number of stairs available at each location and the maximum number of stairs the participant is willing to climb in one go is assessed as well.

The user’s location (GPS coordinates) is collected using Google’s location services that can be linked with the Active2Gether app. The location data are used to determine whether the user visited his or her significant locations (eg, home, study or work place, and sports club) and to derive information about transport and travels that have been made. In addition, information about the characteristics of locations is used for personalized coaching messages to the user. For instance, if a person is being coached on using the stairs more often at their place of work or study, it is only useful to suggest this when the option to climb the stairs is indeed present at the worksite or university.

#### Assessment of Connected Friends

Information regarding the participants’ friends is collected using the Facebook API. Users are asked to provide access to their Facebook ID and their connections by logging into Facebook once and giving permission for this. It is important to note that Facebook does not provide personal information about someone’s Facebook connections but only a list of Facebook IDs of their connections. This information can be used to see whether any Active2Gether users are connected on Facebook. If two participants of the current intervention are connected on Facebook, they see a ranking within the app that shows achievements of both users. In this way, the users only share their achievements with a closed group and not with “everybody,” according to the preferences stated in the focus group discussions.

### Step 5: Developing the Active2Gether Intervention

#### Designing and Developing the Communication Channel

The Active2Gether app shows a nonpersonalized, generic avatar with a welcome message that mentions the user’s current weekly goal. The app displays the current number of daily steps and stairs climbed. In addition, the app shows the following 4 graphs: a bar chart with the step progress toward 70,000 steps per week, a ranking with 6 other Active2Gether users—where possible Facebook friends—based on the step activity over the last seven days, the activity data for each weekday for the current coaching domain (ie, minutes of sport activity, numbers of stair climbed, or minutes of active transport), and the step activity for each weekday. The third and fourth graphs display the user’s own data and the average data assessed within Active2Gether. Moreover, these graphs can be adjusted according to the user’s preferences, that is, they can show data for the last week, last month, or from the first use.

**Figure 3:**
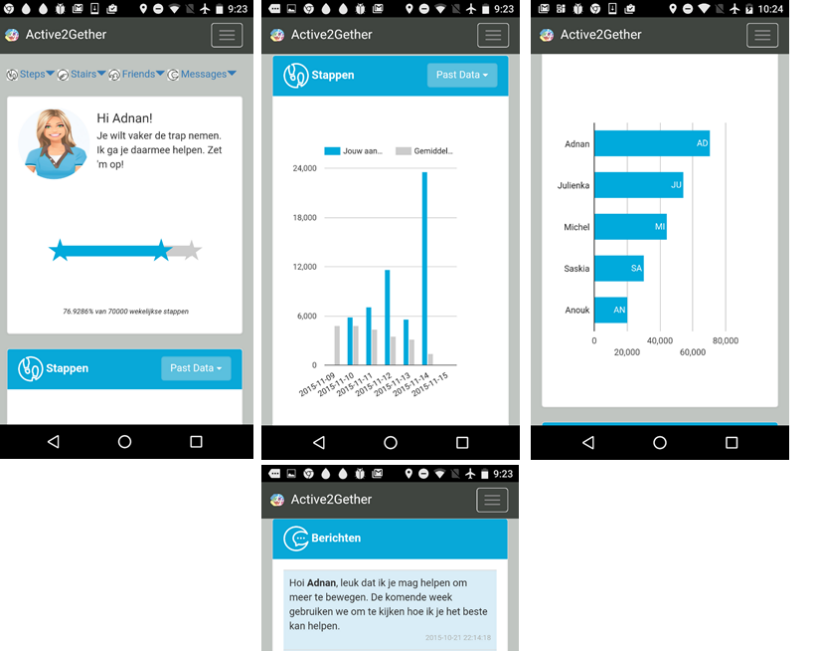
Screenshot of the Active2Gether app.

Tailored messages and short questions are sent via push messages through the app. After the user reads the messages, they are displayed at the bottom of the app. Only the 5 messages sent most recently are displayed in the app. Figure 3 shows a screenshot of the app.

### Step 6: Pilot Testing

The app was adjusted based on the feedback of the 7 participants who pilot-tested the app; for example, the timing of the different steps in the tailoring process (ie, determining the type of feedback, the coaching domain, the weekly goal, and the most promising behavioral determinants) did not originally account for exceptional cases in which a user takes very long to complete a step, causing the next step to be skipped. In the adjusted version, multiple checks and safety mechanisms were implemented to make sure that the tailoring process could still be finished correctly in such conditions. Also, automated messages to remind users to charge their Fitbit and to synchronize their data were added to the system because of the observation that participants in the pilot study sometimes did not notice when it was necessary to do so.

### Step 7: Testing and Evaluating the Intervention

After developing the intervention, an evaluation study was conducted for which data have been collected between March 2016 and September 2016 and data cleaning and initial analyses are now being conducted. A three-arm quasi-experimental trial—with two active control groups—with a baseline and two follow-up assessments at 6 and 12 weeks was conducted to examine the effectiveness of the Active2Gether intervention. This trial is registered in the Dutch trial registry, No. NTR5630. A detailed description of the study protocol can be found in Multimedia Appendix 4 [12,68,69].

## Discussion

This study describes the development and content of Active2Gether, an app-based intervention, which was developed using a systematic and stepwise approach. The aim of the Active2Gether intervention is to empower young adults to become and remain physically active by providing them with app-based tailored coaching and feedback. Active2Gether makes use of an activity tracker and personalized, context-specific feedback. It focuses on 3 PA domains, builds on established behavior theory, and applies evidence-based BCTs and a model-based reasoning system to provide individually tailored coaching messages based on current scores on the behavioral determinants.

The development and content creation of Active2Gether was a stepwise process. The program-planning model proposed by Kreuter et al [41] was mainly used to guide the development and content of the Active2Gether intervention. It states that the health problem needs to be analyzed before developing an intervention, the intervention needs to be based on theory and scientific evidence, and the developmental process is a loop of development, evaluation, and adjustment of the intervention. Program-planning models provide detailed guidance to develop an intervention, which also takes time. Because the possibilities of modern technology in interventions are rapidly evolving, possibilities and preferences that were assessed at the beginning of a lengthy development process may be outdated at the time of implementation or evaluation. The development and content of Active2Gether were guided by relevant health behavior theories and scientific evidence, aiming to develop an intervention that provides a highly tailored feedback. Consequently, less attention was paid to app design and aesthetics that might have resulted in a less appealing app compared with commercial apps. Furthermore, the app is only available for Android devices running on version 4.0 or higher and is therefore not available for older Android devices and smartphones running on other operating systems. Active2Gether incorporates a number of conditions to secure high levels of engagement. First, our approach, integrating a model-based reasoning system, allows us to provide the user with a dynamically tailored intervention that adjusts to the changes in the user. Second, by applying multiple levels of tailoring in the app and the content of the messages (ie, type of support, coaching domain, coaching messages, and weekly goals), the app is likely to be regarded as personally relevant and increase feelings of relatedness. Third, by comparing the PA of the user with that of other Active2Gether users (if possible with their Facebook friends), we expect to further increase personal relevance and relatedness. Lastly, by giving the user the option to select from 3 PA domains and set their own goals with guidance and suggestions based on their own input, we expect higher levels of autonomy, resulting in higher motivation to follow up on the coaching messages. However, to implement these different levels of tailoring, detailed user information is needed repeatedly; thus, frequent user input is needed, which increases user burden.

To date, mobile phones and personal digital assistants have been used to monitor PA with either smartphone apps or external devices, deliver feedback, provide information, and offer a support system to the participants [7]. Active2Gether makes use of an external device, Fitbit One, to monitor PA and provide feedback through the app based on the user’s behavior. However, Active2Gether goes beyond existing interventions by combining data from multiple sources to send context-specific messages. Furthermore, the majority of the published interventions focuses on step activity [70,71], whereas Active2Gether focuses on sports activity, stair walking, and active transport as well. Therefore, the app may be more appealing to participants who do not like to participate in sports, especially because the user can adapt to his or her coaching domain every week. However, Active2Gether does not yet incorporate geofencing (ie, sending location-triggered messages), which would further improve the possibilities for context specificity and real-time feedback and advice by, for example, sending a reminder to climb the stairs at work when users are close to their work location.

So far, the majority of the app-based interventions to promote PA showed positive short-term effects [39]. In line with other app-based PA interventions, Active2Gether makes use of self-monitoring, goal setting, and providing feedback. However, Active2Gether provides dynamically tailored feedback using artificial intelligence-based techniques and including conditional factors (ie, weather), whereas other interventions use logic statements and decision rules to specify which messages should be sent to the user; for example, Active2Gether uniquely assesses behavioral determinants every week to provide tailored advice and feedback on the current behavior, whereas most studies mostly provide feedback on the current behavior only [72-76]. Current app-based interventions to promote PA focus on step activity or overall MVPA [72-77], whereas Active2Gether focuses on sports activities, active transport, and stair walking as well. The majority of papers on app-based interventions reported significant effects [39], and a study that combined machine learning techniques to send personalized messages that were contextualized to the user’s environment and previous behavior showed promising results with regard to the efficacy of the intervention [26]. Because the Active2Gether intervention went beyond the majority of those apps and included BCTs proven to be effective, we expected to find significant intervention effects compared with the 2 (active) control groups.

Active2Gether is ambitious and innovative and incorporates certain risks, for example, the intervention highly relies on input from the activity monitor and location sensor and thus on the user to turn on and synchronize the tracker with the server. Furthermore, it relies on responses from the users on repeated questionnaires. If they do not provide input at all or if they do not provide true and honest answers, the coaching messages that are informed by this information may become irrelevant and nontailored. Moreover, if a participant is not a Facebook user or has no appropriate contacts, the personalization could be limited. Finally, if technical problems are encountered, this may result in errors in synchronization and sending messages late or not at all. To limit the burden for the participants and minimize their input to reduce potential technical problems, future research could make use of smartphone sensors to assess the participant’s behavior.

The overall effectiveness of Active2Gether thus needs to be, and is being, evaluated in a quasi-experimental trial with a 12-week follow-up. However, because app-based interventions offer the possibility to deliver just-in-time interventions that are relevant for the user’s situation for that particular moment, a study is needed to examine the possible effectiveness of specific real-time feedback and advice moments [78]. An ecological momentary assessment [79] in such a quasi-experimental trial setting may help to assess potential specific effects throughout the intervention period. An evaluation of the efficacy of the intervention and the usability can help to further adapt and improve the intervention for future research. Furthermore, data collected during the trial can provide insights on how to further personalize content to the users. The quasi-experimental trial also includes monitoring of app use and a process evaluation of app use and appreciation that will provide information on larger scale dissemination, implementation, and changes required to improve conditions for wider use of the app.

Because the intervention has been developed with an early consideration for the preferences of the target population, it is more likely to meet the expectations of the target population. Consequently, the intervention is more likely to be adopted by the target population. However, the intervention might be prone to technical errors, and a significant input from the user is needed to provide tailored feedback. This might be a burden for the participants, leading to a lower adoption rate. We conducted a small pilot study to test the Active2Gether app and to detect bugs and technical errors; ideally, the pilot study would have been conducted with a larger sample. The current version of the Active2Gether intervention has been developed for young adults with higher education owning a smartphone running on Android version 4.0 or higher. The content needs to be adjusted before offering the intervention to other target populations.

This paper describes the systematic development of an intervention that is based on theory and input from end users. The use of a model-based reasoning system to provide context-specific coaching messages goes beyond many existing eHealth and mHealth interventions.

## Appendix

Multimedia Appendix 1 Overview of the behavior change techniques (BCTs) that were selected to target the behavioral determinants of the theoreticalframework and how they were applied within the intervention.

Multimedia Appendix 2 Components and flow chart of the tailored intervention.

Multimedia Appendix 3 Overview of the questions used for the short and long version of the questionnaire.

Multimedia Appendix 4 Study protocol.

## Abbreviations

API: application programming interface
BCT: behavior change technique
ICT: Information and communications technology
MVPA: moderate-vigorous physical activity
PA: physical activity
SCT: social cognitive theory
