# Supplementary material for: App-Based Intervention Combining Evidence-Based Behavior Change Techniques With a Model-Based Reasoning System to Promote Physical Activity Among Young Adults (Active2Gether): Descriptive Study of the Development and Content
Source: JMIR Res Protoc. 2018 Dec 21;7(12):e185. doi: 10.2196/resprot.7169 (PMC6320419; doi:10.2196/resprot.7169)
Supplement: Multimedia Appendix 1 [file resprot_v7i12e185_app1.pdf]

Multimedia Appendix 1

Overview of the behavior change techniques (BCTs) that were selected to target the behavioral determinants of the theoretical framework and how they were applied within the intervention

Determinant	BCTs	Methods	Parameters for use	How applied	Theory BCT	Ref
**Outcome expectations**	Provide general information on consequences of behavior in general	Belief selection	Requires investigation of the current attitudinal, normative and efficacy beliefs of the individual before choosing the beliefs on which to intervene.	Messages in general and tailored to aspects of the intake questionnaire	Information-motivation-behavioral skills model	[1, 2]
**Self-efficacy**	Action planning/ time management	Guided practice	Subskill demonstration, instruction and enactment with individual feedback	Messages prompting the planning of physical activity, e.g. the suggestion to mark time and day in the calendar	Goal setting theory	[2-5]
	Social comparison	Modeling	Attention, remembrance, skills, reinforcement; credible	Graph tailored to preference social comparison (up-/	Social comparison	[2, 3]

			source, method and channel	downward	theory	
	Persuasion / Problem solving	Verbal persuasion	Credible source.	Persuasive messages on how to overcome barriers	Social cognitive theory	[2, 3]
	Prompt self-monitoring	Self-monitoring of behavior	The monitoring must be of the specific behavior (that is, not of a physiological state or health outcome). The data must be interpreted and used. The reward must be reinforcing to the individual.	Messages that prompt to look at the monitoring graphs and display of graph	Self-regulation, Social cognitive theory, Control theory	[2, 4, 6]
	Plan social support	Mobilizing social networks	Presence of a network that can potentially support health behavior	Messages with suggestions to tell friends and ask for support	Social support theories	[2-4]
	Imaginary reward	Provide contingent rewards/ reinforcement	The reward needs to be tailored to the individual, to follow the behavior in time, and to be seen as a	Messages that tell the user to be proud if they did well	Self-regulation, Social cognitive	[2, 6]

			consequence of the behavior.		theory,; Learning theories	
**Intentions**	Progress towards goal/ Discrepancy between current behavior and goal	Feedback	Feedback needs to be individual, follow the behavior in time, and be specific.	Messages that tell the user how much he/she has already achieved and display of graph	Self-regulation, Social Cognitive Theory	[2-4]
	Motivational messages/verbal persuasion about capacity	Feedback	Feedback needs to be individual, follow the behavior in time, and be specific.	Messages telling the user how well he/she is doing and to keep up the good work or telling the user some advantage of being physically active		[2]
	Modeling/ social comparison	Modeling	Attention, remembrance, skills, reinforcement; credible source, method and channel	Messages stating how well others are doing and display of graph	Social cognitive theory, Theory of planned	[1, 2]

					behavior	
	Provide instruction	Active learning	Time, information and skills	Messages that prompt the user to prepare the sports bag the night before	Social cognitive theory	[1, 2]
	Prompt goal setting	Goal setting	Commitment to the goal; goals that are difficult but available within the individual’s skill level	Messages prompting the user to set a goal and providing a suggestion	Self-regulation, Social cognitive theory	[1, 2]
**Impediments**	Prompt barrier identification / Problem solving	Planning coping responses	Identification of high-risk situations and practice of coping response.	Message that provides information on how to deal with a specific barrier	Social cognitive theory	[1, 2]
**Social norm (descriptive and**	Social comparisons	Graph tailored to preference social comparisons (up-/ downward	Upward comparison may help setting better goals; downward comparison may help feeling more self-	Messages that provide information on how to deal with a specific barrier	Social comparison theory	[1, 2]

**inductive)**			efficacious.			
	Information about others' approval	Information about others' approval	Positive expectations available in social environment	Messages that provide information on how to deal with a specific barrier	Social cognitive theory	[1, 2]
**Self-regulation**	Self-monitoring	Feedback	Feedback needs to be individual, follow the behavior in time, and be specific.	Messages that prompt to look at the monitoring graphs and display of graph	Self-regulation, Social cognitive theory	[1, 2]
	Goal setting	Goal setting	Commitment to the goal; goals that are difficult but available within the individual's skill level	Messages that prompt the user to set a weekly goal	Control theory	[1, 2]
	Progress towards goal/ Discrepancy between current behavior and goal	Feedback	Feedback needs to be individual, follow the behavior in time, and be specific.	Messages that tell the user how much he/she has already achieved and display	Control theory	[1, 2]

				of graph		
	Self-evaluation/ Monitoring outcome(s) of behavior	Self-reevaluation	Stimulation of both cognitive and affective appraisal of self-image. Can use feedback and confrontation; however, raising awareness must be quickly followed by increase in problem-solving ability and self-efficacy.	Messages prompting the user to evaluate how he/she is feeling about failing or achieving the self-set goal	Control theory, Integrated theory of health behavior change	[1, 2]
	Imaginary reward	Provide contingent rewards/reinforcement	The reward needs to be tailored to the individual, to follow the behavior in time, and to be seen as a consequence of the behavior.	Messages that tell the user to be proud if they did well	Self-regulation, Social cognitive theory, Self-determination theory	[1, 2]

**Satisfaction**	Self-evaluation/ Monitoring outcome(s) of behavior	Self-reevaluation	Stimulation of both cognitive and affective appraisal of self-image.<br>Can use feedback and confrontation;<br>however, raising awareness must be<br>quickly followed by increase in problem-solving ability and self-efficacy.	Messages prompting the user to evaluate how he/she is feeling about failing or achieving the self-set goal	Control theory, Integrated theory of health behavior change	[1, 2, 7]
**Long-term goals**	Provide general information on consequences of behavior in general	Consciousness raising	Can use feedback and confrontation;<br>however, raising awareness must be<br>quickly followed by increase in problem-solving ability and (collective) self-efficacy.	Messages providing general information on consequences of behavior in general	Information-motivation-behavioral skills model	[1, 2]

1. Abraham C, Michie S. A taxonomy of behavior change techniques used in interventions. Health Psychol. 2008 5/2008;27(3):379-87. doi: 2008-08834-010 [pii];10.1037/0278-6133.27.3.379 [doi].
2. Kok G, Gottlieb NH, Peters GJ, Mullen PD, Parcel GS, Ruiter RA, et al. A taxonomy of behaviour change methods: an Intervention Mapping approach. Health Psychol Rev. 2016 Sep;10(3):297-312. PMID: 26262912. doi: 10.1080/17437199.2015.1077155.
3. Williams SL, French DP. What are the most effective intervention techniques for changing physical activity self-efficacy and physical activity behaviour--and are they the same? Health Educ Res. 2011 4/2011;26(2):308-22. doi: cyr005 [pii];10.1093/her/cyr005.
4. Olander EK, Fletcher H, Williams S, Atkinson L, Turner A, French DP. What are the most effective techniques in changing obese individuals' physical activity self-efficacy and behaviour: a systematic review and meta-analysis. Int J Behav Nutr Phys Act. 2013 2013;10:29. doi: 1479-5868-10-29 [pii];10.1186/1479-5868-10-29.
5. Plotnikoff RC, Costigan SA, Karunamuni N, Lubans DR. Social cognitive theories used to explain physical activity behavior in adolescents: a systematic review and meta-analysis. Prev Med. 2013 May;56(5):245-53. PMID: 23370047. doi: 10.1016/j.ypmed.2013.01.013.
6. Sniehotta FF. Towards a theory of intentional behaviour change: plans, planning, and self-regulation. Br J Health Psychol. 2009 May;14(Pt 2):261-73. PMID: 19102817. doi: 10.1348/135910708X389042.
7. Abraham C, Michie S. Coding Manual to Identify Behaviour Change Techniques in Behaviour Change Intervention Descriptions. 2007 [updated 2007; cited 2013 1/7/2013]; Available from: http://www.webcitation.org/6ITFkuVgk.
