# Supplementary figures and images for: App-Based Intervention Combining Evidence-Based Behavior Change Techniques With a Model-Based Reasoning System to Promote Physical Activity Among Young Adults (Active2Gether): Descriptive Study of the Development and Content
Source: JMIR Res Protoc. 2018 Dec 21;7(12):e185. doi: 10.2196/resprot.7169 (PMC6320419; doi:10.2196/resprot.7169)

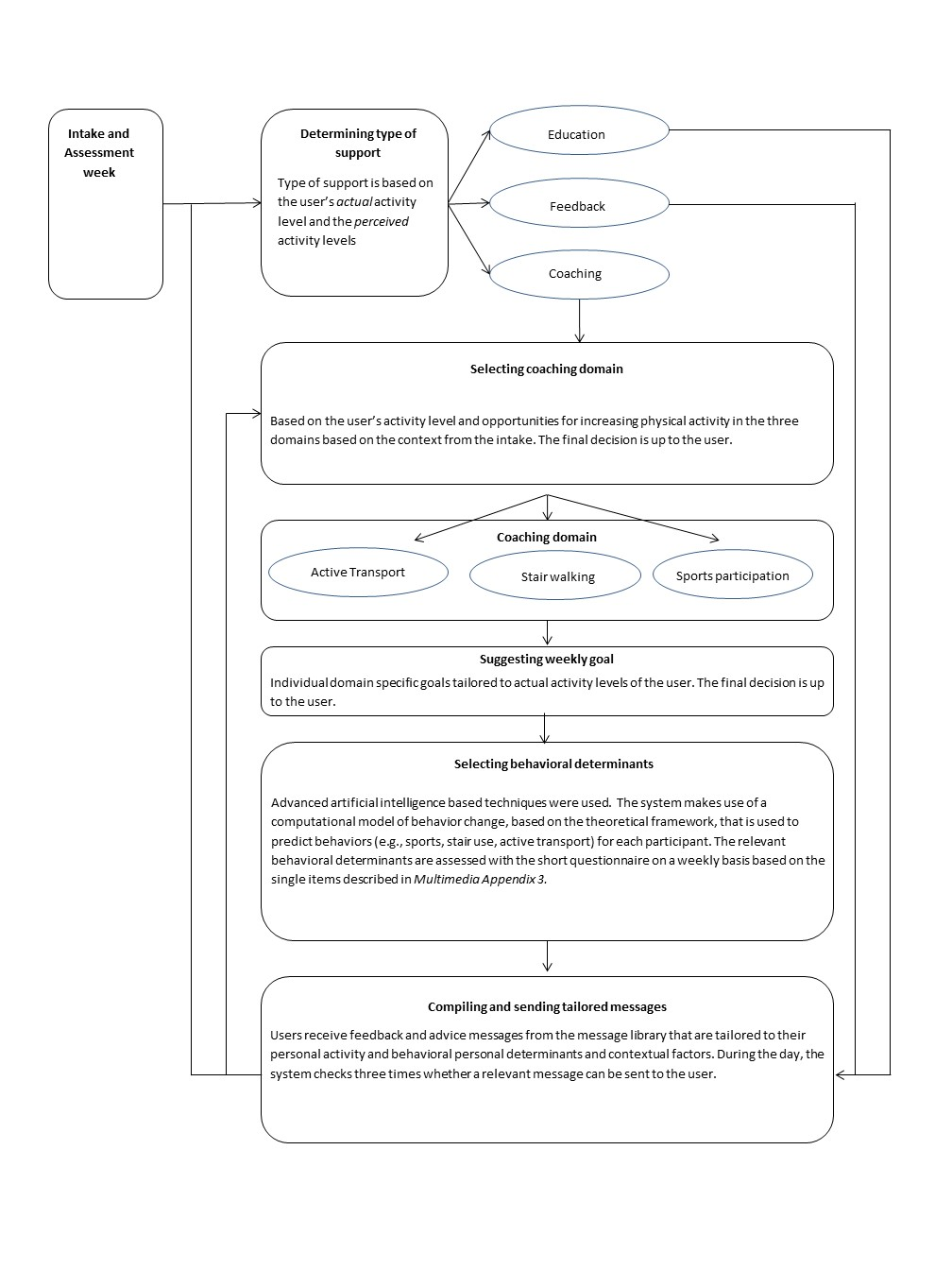

Supplement: Multimedia Appendix 2 [file resprot_v7i12e185_app2.png]
