# Supplementary material for: App-Based Intervention Combining Evidence-Based Behavior Change Techniques With a Model-Based Reasoning System to Promote Physical Activity Among Young Adults (Active2Gether): Descriptive Study of the Development and Content
Source: JMIR Res Protoc. 2018 Dec 21;7(12):e185. doi: 10.2196/resprot.7169 (PMC6320419; doi:10.2196/resprot.7169)
Supplement: Multimedia Appendix 3 [file resprot_v7i12e185_app3.pdf]

Behavior determinant	Example question long version	Number of items long version	Question short version	Answer possibilities
Outcome Expectations [NQLS[1], [68]]	**If I participate in regular physical activity or sports, then:**<br>- I will improve my health<br>- I will feel more attractive<br>- I will lose weight<br>- I will improve my physical fitness<br>- I will feel relaxation<br>- I will feel less tension and stress	6	If I participate in regular **sports**, then it will have a positive effect, for example on my healthy, appearance, weight or how I will feel.<br><br>If I regularly **take the stairs**, then it will have a positive effect, for example on my healthy, appearance, weight or how I will feel.<br><br>If I regularly **bike or walk**, then it will have a positive effect, for example on my healthy, appearance, weight or how I will feel.	1 – No reason at all<br>2 – A slightly important reason<br>3 – A quite important reason<br>4 – A very important reason

Self-efficacy [Self-efficacy scales for exercise [79], NQLS[1] [68]]	**How confident are you that you could do PA, in each of the following situations? I'm confident that I could:**<br>- Do PA even when I'm tired<br>- Do PA even when I'm in a bad mood<br>- Do PA even when I feel I don't have time<br>- Do PA even when I am on holiday<br>- Do PA even when it is raining	12	How confident are you that you will do sports in the next week even when you're tired, busy or when it's bad weather?<br><br>How confident are you that you will take the stairs in the next week even when you're tired, you're in a hurry or you're with others?<br><br>How confident are you that you will cycle or walk to work/the university in the next week, even when you're tired, you're busy or when it's bad weather?	1 – Not at all confident<br>2 – Slightly confident<br>3 – Moderate Confident<br>4 – Very Confident<br>5 – Extremely confident

Perceived barriers for sport [NQLS[1] [68]]	**How often do the following barriers prevent you from doing sports activities?**<br>- Bad weather<br>- Lack of time<br>- Lack of interest in exercise<br>- Other priorities<br>- Lack of skills or knowledge<br>- Lack of equipment<br>- Lack of facilities or space<br>- Lack of physical fitness<br>- Lack of energy<br>- Lack of money<br>- Lack of company<br>- Self-conscious about my looks when I exercise	12	How often do barriers prevent you from participating in sports or exercise activities? Think for example of lack of time, lack of energy, costs, lack of company.	1 – Never<br>2 – Rarely<br>3 – Sometimes<br>4 – Often<br>5 – Very often
Perceived barriers for active transport	**How often do the following barriers prevent you from traveling by bike or by walking instead of traveling by car or public transport?**<br>- Bad weather<br>- Lack of time<br>- Lack of physical fitness<br>- Lack of energy<br>- Too many pieces of luggage<br>- Travel distance is too far away<br>- No suitable bike	7	How often do barriers prevent you from **cycling or walking** to work /the university instead of traveling by public transport or car? Think for example of lack of time, lack of physical fitness, lack of energy or too many pieces of luggage.	1 – Never<br>2 – Rarely<br>3 – Sometimes<br>4 – Often<br>5 – Very often

Perceived barriers for stairs climbing	**How often do the following barriers from climbing the stairs?**<br>- Lack of physical fitness<br>- Lack of energy<br>- Too many pieces of luggage<br>- Too many flights of stairs	4	How often do barriers prevent you from climbing the stairs? For example barriers as being in a hurry, lack of physical fitness, lack of energy or carrying too many pieces of luggage?	1 – Never<br>2 – Rarely<br>3 – Sometimes<br>4 – Often<br>5 – Very often
Social norm descriptive	- My friends think that I should be sufficient physically active.<br>- My fellow students think that I should be sufficient physically active.<br>- My brother(s) and/or sister(s) think that I should be sufficient physically active.	3	For the people around me (friends, fellow students, family), it's important that I sufficiently participate in sports.<br><br>For the people around me (friends, fellow students, family), it's important that I use the stairs instead of the elevator.<br><br>For the people around me (friends, fellow students, family), it's	1 – I strongly disagree<br>2 – I somewhat disagree<br>3 – Neutral<br>4 – I somewhat agree<br>5 – I strongly agree

			important that I regularly bike or walk to work/the university.	
Social norm injunctive	- How often do your friends/roommates/ brothers or sisters/ parents participate in physical activities	4	Not applicable[2]	1 – Never<br>2 – Rarely<br>3 – Sometimes<br>4 – Often<br>5 – Very often<br>6 – Not applicable
Intention	Do you intend do sports (more often) within the next week/month/6 months even if you think you're already sufficiently active?	3	I intend to do sports (more often) within the next week.<br><br>I intend to climb the stairs (more often) within the next week.<br><br>I intend to bike or walk to work/the university (more often) within the next week.	1 – Most definitely will not<br>2 – Probably will not<br>3 – Maybe / maybe not<br>4 – Probably will<br>5 – Most definitely will

Self-regulation [12] [Anderson et al, 2006 [12]	I keep track of how active I am.<br>I check whether I met my goals<br><br>In the last three months I:<br>- Set aside time for my daily physical activity<br>- Walked or biked instead of drove or travelled by public transport<br>- I exercised or did physical activities with someone else<br>- I wrote it down in my calendar to do sports/physical activity<br>- I planned do sports/exercise even when the weather was bad	7	I planned and set goals do regular sports/exercise.<br>When I wasn't able do it I evaluated why I wasn't able to do it and whether I needed to change something.	1 – Never<br>2 – Rarely<br>3 – Sometimes<br>4 – Often<br>5 – Very often
Satisfaction	How satisfied are you about how physically active you are?	1	How satisfied are you about how often you did sports in the last week?<br><br>How satisfied are you about how often you	0 – Very unhappy<br><br>10 – Very happy

			climbed the stairs last week?<br><br>How satisfied are you about how often you biked or walked to work/the university last week?	
Long-term goals	Not applicable[3]	0	How motivated are you to be more physically active?	0 – Very unmotivated<br><br>10 – Very motivated

[1] NQLS= Neighborhood Quality of Life Study

[2] Questions assessing social norm injunctive were not included in the short questionnaire.

[3] Long-term goals were not included in the long questionnaire
