# Supplementary material for: App-Based Intervention Combining Evidence-Based Behavior Change Techniques With a Model-Based Reasoning System to Promote Physical Activity Among Young Adults (Active2Gether): Descriptive Study of the Development and Content
Source: JMIR Res Protoc. 2018 Dec 21;7(12):e185. doi: 10.2196/resprot.7169 (PMC6320419; doi:10.2196/resprot.7169)
Supplement: Multimedia Appendix 4 [file resprot_v7i12e185_app4.pdf]

Study protocol

Study design

A three-arm quasi-experimental trial with a baseline and two follow-up assessments at 6 and 12 weeks is being conducted to examine the effectiveness of the Active2Gether intervention. This trial is registered in the Dutch trial registry, No. NTR5630.

The three study arms are two versions of Active2Gether and an active control group. The first version of Active2Gether is the full version with personalized coaching messages for the three coaching domains, weekly goals, self-monitoring and social comparison, as described in Step 1-3. The second version of Active2Gether is similar, but does not include the coaching part, i.e., selecting a coaching domain, setting a weekly goal, receiving personalized messages to increase levels of PA. The second version of Active2Gether does still allow the users to monitor their step activity and to compare themselves with other participants participating in the trial. The control condition will receive the Fitbit One activity tracker and the Fitbit app, i.e. an existing app-based intervention with much lower levels of tailored feedback and advice. The comparison between the two Active2Gether versions will provide information regarding the effects, use and appreciation of the coaching part of Active2Gether, the comparison with the Fitbit control arm will provide information about the effects, use and appreciation as compared to an existing 'usual care' PA monitoring and promotion device.

Hypotheses

The null hypothesis is that there is no difference in mean daily minutes of moderate-vigorous physical activity (MVPA) between the three intervention groups, i.e. Active2Gether Full, Active2Gether Light and Fitbit, at 12-week follow up. The alternative hypothesis is that the Active2Gether Full condition will show significantly higher levels of mean daily minutes of MVPA in comparison to the Active2Gether Light and Fitbit condition at 12-week follow up.

Recruitment and Participants

Participants (N = 160-200) that are representative for the target population, i.e. young adults between 18 and 30 years, are recruited by flyers, posters, social media and personal contacts, and snow ball strategies in the Amsterdam and Utrecht regions.

Participants are then asked to sign up for the trial through the Active2Gether website. For stratification purposes during the randomization process, the participants are asked to provide information about sex, age and type of smartphone they use (i.e., iOS or Android).

Stratified randomization is applied based on the type of smartphone and gender. As the A2G app only runs on Android, iPhone owners will automatically be assigned to the Fitbit condition. Android users will be assigned to the Fitbit condition when possible. The aim is to divide men and women with an Android phone equally over the two A2G conditions. This was done by using a 1:1 ratio applied to the order of registering.

Participants are eligible for this study when they meet the following criteria: (a) aged 18-30 years; (b) being (apparently) healthy; (c) Dutch speaking and (d) signed the informed consent form, (e) have a suitable smartphone running on iOS or Android. The study has been approved by the Medical Ethical Committee of the VU Medical Center (2015.363).

Measures

Physical Activity

PA is assessed with two types of tri-axial accelerometers. The ActiGraph wGT3x-BT is used to evaluate the efficacy – thus mean daily minutes of MVPA – of the intervention and will be assessed at baseline and 12 weeks follow-up for one week. Furthermore, all participants will receive a Fitbit One that is used to continuously monitor their PA behavior in minute-by-minute intervals and allows the participants to monitor their PA behavior for 12 weeks.

Behavioral determinants

To evaluate changes in behavior determinants, participants are asked to fill in a questionnaire assessing all behavioral determinants that are in the theoretical framework at baseline, 6 and 12 weeks follow-up. The questionnaire is based on existing questionnaires that have previously been validated [1-3]

App usage and appreciation

Information regarding the use and appreciation of the app - the two versions of the Active2Gether app and the Fitbit app - is collected at 12 weeks follow-up. During the trial, additional qualitative research is conducted to explore the users' experiences to improve the Active2Gether intervention. A subgroup of participants that use the Active2Gether app including the coaching part are asked for an interview capturing their experiences with the app. Lastly, for both Active2Gether apps information about the number of app-log-ins are collected as well.

App use and dropouts

Engagement with the intervention is assessed using the number of coaching messages read – only for the A2G-Full condition - and Fitbit usage (for all participants). As all participants are asked to wear the Fitbit during the intervention, the number of valid days the Fitbit was worn during the 12 weeks – thus 84 days – can be used as an indicator for attrition.

User experience

User appreciation is assessed with 20 items using a 7-point Likert Scale. Examples of statements in this assessment tool are: (1) satisfaction: "the app meets my expectations", (2) user friendliness: "I can easily find the information I'm looking for", (3) perceived effectiveness: "the app motivates me to achieve my goals", and (4) professionality: "the app looks professional".

Evaluation of questions and messages (A2G)

Evaluation of the number of questions (Active2Gether-Full and Light) and messages (Active2Gether-Full) sent to the respondent by the app is assessed on 5-point Likert scales asking, 'the number of questions/messages sent by the app are…'. Additionally, participants in the Active2Gether Full condition receive eight statements on: a) tone (i.e., how friendly is the message written), b) authority (i.e., the message is an obligation to do something), c) personal relevancy, d) trustworthiness, e) motivational strength, f) empathy.

Positive and negative aspects

Participants could list up to three positive and three negative features of the app in a free text.

Analyses

The trial data will be analyzed for differences in effects of the two versions of Active2Gether as compared to the Fitbit app. The primary outcomes will be total MVPA. Secondary outcomes will be changes in determinants of PA, i.e. self-efficacy, outcome expectations, intention, self-regulation, social norm, long-term goals, and satisfaction.

The efficacy of the intervention will be assessed in multiple steps. First, linear regression analyses will be used to compare MVPA at follow-up, adjusted for baseline values, between the different conditions. Second, secondary outcome measures will also be compared across the three conditions. Thus the effectiveness of the intervention to change weekly minutes spent in sports activities, number of stairs climbed, and minutes of active transport will be evaluated as well. Third, the hypothesized underlying processes will be evaluated using mediation analysis. With mediation

analysis we will examine whether the intervention successfully changes the addressed behavioral determinants and whether that in turn resulted in changes in MVPA.

Usability and levels of engagement will be evaluated, by analyzing the duration and frequency of involvement with the Active2Gether app, and indicators of non-usage attrition.

References

1. Anderson ES, Wojcik JR, Winett RA, Williams DM. Social-cognitive determinants of physical activity: the influence of social support, self-efficacy, outcome expectations, and self-regulation among participants in a church-based health promotion study. Health Psychol. 2006 7/2006;25(4):510-20. doi: 2006-08842-008 [pii];10.1037/0278-6133.25.4.510.

2. Sallis JF, Pinski RB, Grossman RM, Patterson TL, Nader PR. The development of self-efficacy scales for healthrelated diet and exercise behaviors. Health Education Research; 1988. p. 283-92.

3. The Neighborhood Quality of Life Study The Neighborhood Quality of Life Study (NQLS) Survey. 2001 [07-05-2017]; Available from: http://sallis.ucsd.edu/Documents/Measures_documents/NQLS_S1.pdf.
